# USING PHOTOVOICE TO UNDERSTAND THE LIVED EXPERIENCE AND ENVIRONMENT OF OLDER ADULTS AWAITING KIDNEY TRANSPLANT

**DOI:** 10.1093/geroni/igac059.1493

**Published:** 2022-12-20

**Authors:** Melissa Hladek, Deborah Wilson, Meera Shanbhag, Katie Krasnansky, Jeremy Walston, Qian-Li Xue, Mara McAdams-DeMarco, Sarah Szanton

**Affiliations:** Johns Hopkins University, Baltimore, Maryland, United States; Johns Hopkins University School of Nursing, Baltimore, Maryland, United States; Johns Hopkins University, Baltimore, Maryland, United States; Johns Hopkins University, Baltimore, Maryland, United States; Johns Hopkins University, Baltimore, Maryland, United States; Johns Hopkins University, Baltimore, Maryland, United States; NYU Grossman School of Medicine and NYU Langone Health, Baltimore, Maryland, United States; Johns Hopkins University School of Nursing, Baltimore, Maryland, United States

## Abstract

End-stage renal disease (ESRD) causes age-accelerating disease and disproportionally affects older and minority patients; the preferred treatment is kidney transplantation (KT). Rates of KT have risen 5-fold for older adults since 1990 but there is great heterogeneity in KT outcomes by frailty and waitlist status. The person-environment fit theory posits that improving a person’s lived environment will facilitate optimal individual functioning. We recruited a balanced sample of 20 participants (mean age 60, range 35-82) with respect to their frail/ pre-frail and inactive/ active waitlist status to explore personal experience and the lived environment through the use of photovoice, gaining insight into the person-environment fit of those older adults awaiting KT. We will discuss IRB issues, recruitment and logistical tips with using Photovoice. This work will help to inform what vulnerable and marginalized older adults awaiting KT must be prepared for psychologically and physically, including modifications needed to their lived environments.

